# 319. Clinical accuracy and utility of plasma microbial cell free DNA NGS for the diagnosis of invasive aspergillosis in patients with hematologic malignancy and COVID-19

**DOI:** 10.1093/ofid/ofac492.397

**Published:** 2022-12-15

**Authors:** Ki Hyun Lee, Joon-sup Yeom, Jun Yong Choi, Nam Su Ku, Su Jin Jeong, Jung Ho Kim, Se Ju Lee, Jinnam Kim, Chang Hyup Kim, Jung Ah Lee, Ji Eun Jang, Dongju Won, Saeam Shin, Seung-Tae Lee, Jin Young Ahn

**Affiliations:** Yonsei University College of Medicine, Seoul, Seoul-t'ukpyolsi, Republic of Korea; Division of Infectious Diseases, Department of Internal Medicine, Yonsei University College of Medicine, Seoul, Seoul-t'ukpyolsi, Republic of Korea; Yonsei University College of Medicine, Seoul, Seoul-t'ukpyolsi, Republic of Korea; Division of Infectious Diseases, Department of Internal Medicine, Yonsei University College of Medicine, Seoul, Seoul-t'ukpyolsi, Republic of Korea; Yonsei University College of Medicine, Seoul, Seoul-t'ukpyolsi, Republic of Korea; Yonsei University College of Medicine, Seoul, Seoul-t'ukpyolsi, Republic of Korea; Yonsei University College of Medicine, Seoul, Seoul-t'ukpyolsi, Republic of Korea; Yonsei University College of Medicine, Seoul, Seoul-t'ukpyolsi, Republic of Korea; Yonsei University College of Medicine, Seoul, Seoul-t'ukpyolsi, Republic of Korea; Yonsei University College of Medicine, Seoul, Seoul-t'ukpyolsi, Republic of Korea; Yonsei University College of Medicine, Seoul, Seoul-t'ukpyolsi, Republic of Korea; Yonsei University College of Medicine, Seoul, Seoul-t'ukpyolsi, Republic of Korea; Yonsei University College of Medicine, Seoul, Seoul-t'ukpyolsi, Republic of Korea; Yonsei University College of Medicine, Seoul, Seoul-t'ukpyolsi, Republic of Korea; Yonsei University College of Medicine, Seoul, Seoul-t'ukpyolsi, Republic of Korea

## Abstract

**Background:**

Invasive aspergillosis (IA) is a great threat to the severely immunocompromised and patients with coronavirus disease (COVID-19). However, diagnosis of IA is often difficult due to need for invasive biopsy and low sensitivity of other diagnostic tests. Next-generation sequencing (NGS) of plasma cell free DNA (cfDNA) can be a novel non-invasive diagnostic modality. We evaluated the clinical accuracy and utility of microbial cfDNA NGS for the diagnosis of IA in patients with hematologic malignancy (HM) and COVID-19.

**Methods:**

A single-center prospective study of plasma microbial cfDNA NGS was conducted in a tertiary-care hospital in South Korea. We enrolled adult patients with HM and COVID-19, who suspected IA and performed conventional diagnostic tests for IA. The results of NGS were compared with the diagnosis of IA through conventional methods. IA cases were diagnosed according to EORTC/MSG definitions in patients with HM, and modified AspICU criteria in patients with COVID-19. (Figure 1).

**Figure 1.**
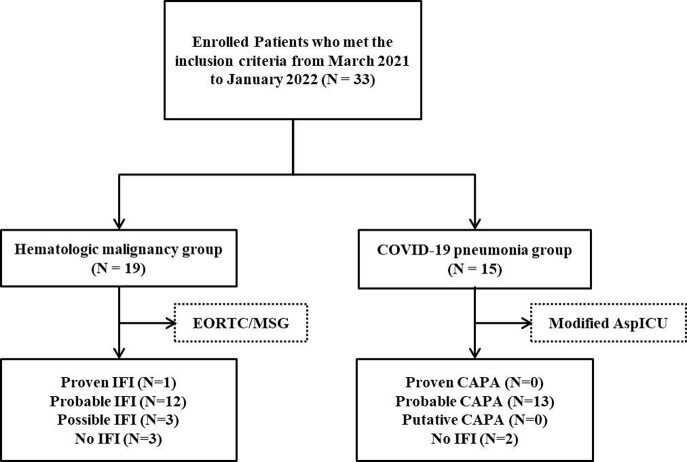
Flow chart for the participant selection method used in this study

**Results:**

Between March 2021 and January 2022, a total of 33 participants (22 [64.7%] male, median age 66.0 [50.5, 72.0]) were enrolled;19 participants with HM and 15 with COVID-19 were analyzed (Figure1 and Table1). In participants with HM, aspergillus cfDNA was detected in 100% of both proven (1/1) and probable (12/12) IA cases, and 33.3% of both possible (1/3) and no IA (1/3) cases. In participants with COVID-19, 46.2% of probable IA (6/13) showed positive aspergillus cfDNA. Detection rate of aspergillus cfDNA was significantly higher in proven/probable IA cases in participants with HM compared to participants with COVID-19. (100% vs 46.2%, p=0.005) (Figure 2). As shown in Table 2, among proven/probable IA cases, participants with positive aspergillus cfDNA showed significantly higher rate of having uncontrolled hematologic disease, receiving stem cell transplantation and recent chemotherapy. In three participants with HM, non-aspergillus strains confirmed by cfDNA NGS were in accordance with pathogens identified through conventional culture methods.

**Table 1.**
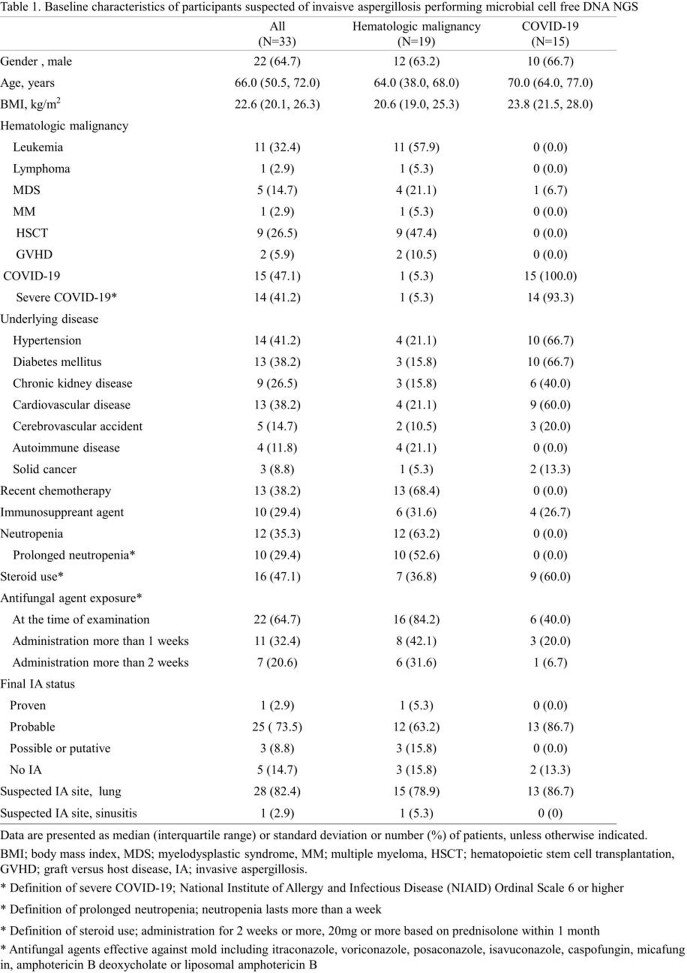
Baseline characteristics of participants suspected of invaisve aspergillosis performing microbial cell free DNA NGS

**Figure 2.**
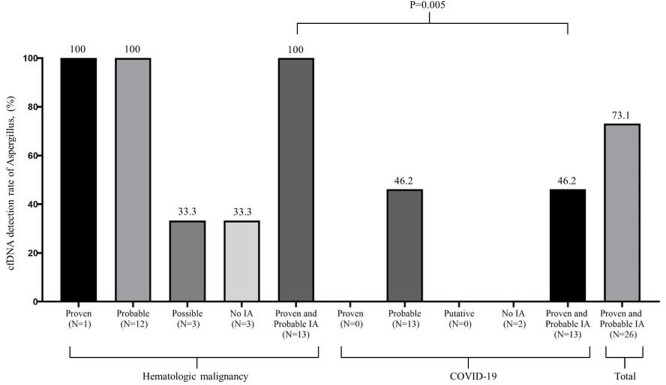
cfDNA detection rate in participants with suspected fungal infection according to the EORTC/MSG or modified AspICU diagnostic criteria

**Figure:**
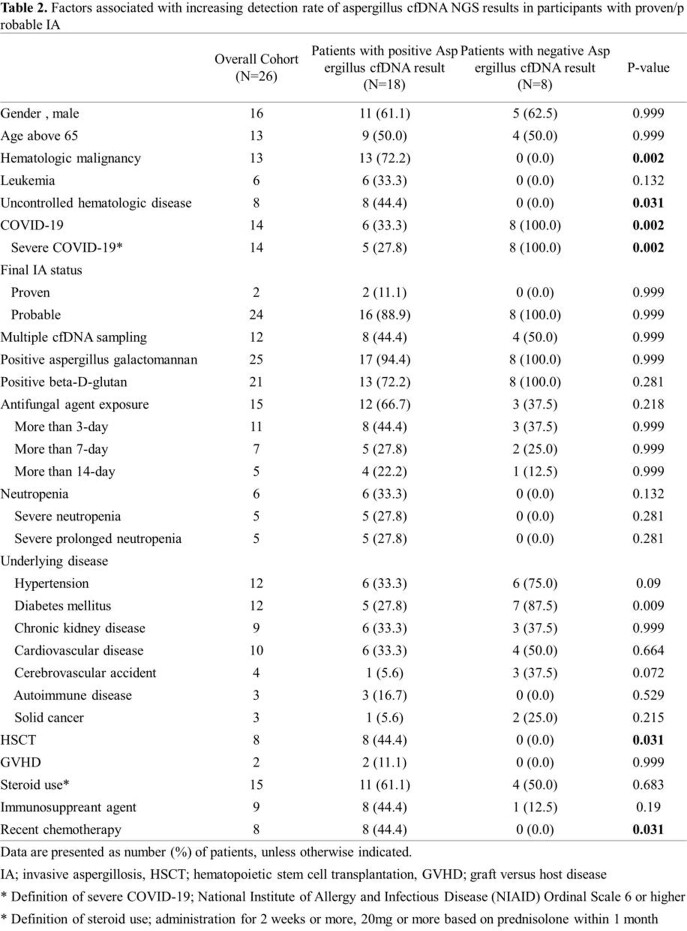

**Conclusion:**

Detection of aspergillus cfDNA showed high concordance in the results of conventional diagnostic methods in proven/probable IA of patients with HM and could be a helpful non-invasive approach to IA diagnosis in those populations.

**Disclosures:**

**All Authors**: No reported disclosures.

